# Supplementary material for: Cranial Ultrasound Abnormalities in Small for Gestational Age or Growth-Restricted Infants Born over 32 Weeks Gestation: A Systematic Review and Meta-Analysis
Source: Brain Sci. 2022 Dec 14;12(12):1713. doi: 10.3390/brainsci12121713 (PMC9776358; doi:10.3390/brainsci12121713)
Supplement: Supplementary file 1 [file brainsci-12-01713-s001.zip › brainsci-2016742-supplementary.pdf]

Appendix SA

Search Terms

PubMed Search Terms:

(((((brain ultrasound[MeSH Terms])) OR (cranial ultrasound[MeSH Terms])) OR (echoencephalography[MeSH Terms]))) OR ((intracranial hemorrhage[MeSH Terms])) OR (periventricular leucomalacia[MeSH Terms]) OR (intraventricular haemorrhage) OR (intraventricular hemorrhage) OR (intracranial pathology) OR (intracranial haemorrhage) OR (intracerebral haemorrhage) OR (intracerebral hemorrhage) AND ((((((fetal growth retardation[MeSH Terms]) OR (retardation, intrauterine growth[MeSH Terms])) OR (infant, small for gestational age[MeSH Terms])) OR ("growth restriction")) OR ("fetal growth restriction")) OR ("intrauterine growth restriction")) AND ((humans[Filter]) AND (english[Filter]))

MEDLINE Search Terms:

1 Infant, Newborn/ or Infant, Premature/ or Obstetric Labor, Premature/ or Premature Birth/

2 (Late Pre?term or moderate Pre?term or pre?mature* or term or neonate* or newborn*).tw.

3 Infant, Low Birth Weight/ or "Infant, Small for Gestational Age"/ or Infant, Very Low Birth Weight/

4 ((infant* or neonate*newborn*) adj3 (low birth weight or small for gestational age)).tw.

5 *Fetal Growth Retardation/ or *Infant, Premature, Diseases/ or Intensive Care, Neonatal/ or Intensive Care Units, Neonatal/

6 (((fetal or foetal) adj (growth retardation or growth restriction)) or FGR).tw.

7 ((intrauterine adj (growth retardation or growth restriction)) or IUGR or 'growth restriction').tw.

8 Echoencephalography/ or Neonatal Screening/ or cranial ultrasound.tw. or CUS.tw. or brain ultrasound.tw.

9 *Cerebellar Hemorrage/ or *Cerebral Hemorrhage/ or *Cerebral Intraventricular Hemorrhage/ or *Leu?omalacia, Periventricular/

10 ((h?emorrhage adj (intraventricular or periventricular or intracerebral or cerebellar)) or IVH or PIVH or periventricular leu?omalacia or PVL or intracranial h?emorrhage or intracranial pathology or intracerebral h?emorrhage).tw.

11 1 or 2

12 3 or 4 or 5 or 6 or 7

13 9 or 10

14 8 and 13

15 11 and 12 and 14

16 limit 15 to humans

EMBASE Search Terms:

1 Infant, Low Birth Weight/ or "Infant, Small for Gestational Age"/ or Infant, Very Low Birth Weight/

2 ((infant* or neonate*newborn*) adj3 (low birth weight or small for gestational age)).tw.

3 *Fetal Growth Retardation/ or *Infant, Premature, Diseases/ or Intensive Care, Neonatal/ or Intensive Care Units, Neonatal/

4 (fetal growth retardation or fetal growth restriction or FGR).tw.

5 (intrauterine growth retardation or intrauterine growth restriction or IUGR).tw.

6 1 or 2 or 3 or 4 or 5

7 Echoencephalography/ or cranial ultrasound.tw. or CUS.tw. or brain ultrasound.tw.

8 (Echoencephalography/ or cranial ultrasound.tw. or CUS.tw. or brain ultrasound.tw.) and (abnormalit* or defect*).tw.

9 *Cerebellar Hemorrage/ or *Cerebral Hemorrhage/ or *Cerebral Intraventricular Hemorrhage/ or *Leu?omalacia, Periventricular/

10 ((h?emorrhage adj (intraventricular or periventricular or intracerebral or cerebellar or intracranial)) or IVH or PIVH or periventricular leu?omalacia or PVL or intracranial pathology).tw.

11 7 and 9

12 7 and 10

13 7 or 8 or 11 or 12

14 6 and 13

15 limit 14 to human

Appendix SB

THE COCHRANE COLLABORATION®

Data collection form

Intervention review – RCTs and non-RCTs

Adopted from: Effective Practice and Organisation of Care (EPOC). Data collection form. EPOC Resources for review authors. Oslo: Norwegian Knowledge Centre for the Health Services; 2013. Available at: http://epoc.cochrane.org/epoc-specific-resources-review-authors

**Review title or ID**

**Study ID** *(surname of first author and year first full report of study was published e.g. Smith [illegible]*

**Report IDs of other reports of this study** *(e.g. duplicate publications, follow-up studies)*

General Information

1. **Date form completed**	
2. **Name/ID of person extracting data**	
3. **Report title**	
4. **Reference details**	
5. **Report author contact details**	
6. **Publication type**	
7. **Study funding source**	
8. **Possible conflicts of interest**	
9. **Notes:**	

Eligibility

**Study Characteristics**	**Review Inclusion Criteria**	**Yes/ No / Unclear**	**Location in text** *(pg & ¶/fig/table)*
10. **Type of study**	Case Control	…	
	Cohort Study	…	

Study Characteristics	Review Inclusion Criteria	Yes/ No / Unclear	Location in text *(pg & ¶/fig/table)*
	Descriptive Study	…	
	Other design (specify):<br>•	…	
11. **Participants**	…	…	
12. **Subgroup analysis present for gestation**		…	
13. **Subgroup analysis present for weight criteria- SGA/FGR**			
14. **Cranial ultrasound performed postnatally**			
15. **Decision:**			
16. **Reason for exclusion**			

DO NOT PROCEED IF STUDY EXCLUDED FROM REVIEW

Population and setting

	Description	Location in text *(pg & ¶/fig/table)*
17. **Population description**		
18. **Setting**		
19. **Inclusion criteria**		
20. **Exclusion criteria**		
21. **Method/s of recruitment of participants**		

Methods

	Descriptions as stated in report/paper	Location in text *(pg & ¶/fig/table)*
22. **Aim of study**		
23. **Design**		
24. **Start date**		
25. **End date**		

Risk of Bias assessment

Domain	Risk of bias *Low/ High/Unclear*	Support for judgement	Location in text *(pg & ¶/fig/table)*
26. **Incomplete outcome data**	…		
27. **Selective outcome reporting?**	…		
28. **Other bias**	…		

Participants

Provide overall data and, if available, comparative data for each intervention or comparison group.

	Description as stated in report/paper	Location in text (pg & ¶/fig/table)
29. **Total no. infants included**		

	Description as stated in report/paper	Location in text (pg & ¶/fig/table)
30. **Withdrawals and exclusions** *(if not provided below by outcome)*		
31. **Gestation included**		
32. **Gestation relevant to subgroup analysis**		
33. **Sex**		
34. **Co-morbidities**		
35. **Subgroups measured**		
36. **Subgroups reported**		
37. **Notes:**		

Outcomes

	Description as stated in report/paper	Location in text *(pg & ¶/fig/table)*
38. **Outcome name**	Cranial ultrasound abnormalities	
39. **Time points measured**		
40. **Time points reported**		
41. **Outcome definition**		
42. **Person performing imaging**		
43. **Person reporting imaging**		

Results

	Description as stated in report/paper				Location in text *(pg & ¶/fig/table)*
44. **Comparison**					
45. **Outcome**					
46. **Subgroup (with definition)**					
47. **Results**	**FGR/SGA Infants**		**AGA Infants**		
	No. events	No. participants	No. events	No. participants	

	Description as stated in report/paper		Location in text *(pg & ¶/fig/table)*
48. **No. missing participants and reasons**			
49. **Key findings of study**			
50. **Statistical methods used and appropriateness of these methods**			

Applicability

51. **Have important populations been excluded from the study?**	… *Yes/No/Unclear*	
52. **Does the study directly address the review question?**	… *Yes/No/Unclear*	
53. **Notes:**		

Other information

	Description as stated in report/paper	Location in text *(pg & ¶/fig/table)*
54. **Key conclusions of study authors**		
55. **References to other relevant studies**		
56. **Correspondence required for further study information**		
57. **Further study information requested**		
58. **Correspondence received**		

Appendix SC

Newcastle Ottawa Scale

COHORT STUDIES

Note: A study can be awarded a maximum of one star for each numbered item within the Selection and Outcome categories. A maximum of two stars can be given for Comparability

Selection

1. Representativeness of the exposed cohort
    a. truly representative of the average neonatal population ★
    b. somewhat representative of the average neonatal population ★
    c. selected group of users eg nurses, volunteers
    d. no description of the derivation of the cohort
2. Selection of the non exposed cohort
    a. drawn from the same community as the exposed cohort ★
    b. drawn from a different source
    c. no description of the derivation of the non exposed cohort
3. Ascertainment of exposure
    a. secure record (eg surgical records) ★
    b. structured interview ★
    c. written self report
    d. no description
4. Demonstration that outcome of interest was not present at start of study
    a. yes ★
    b. no

Comparability

1. Comparability of cohorts on the basis of the design or analysis
    a. study controls for weight ★
    b. study controls for any additional factor ★ FGR vs SGA, steroid use, chorioamnionitis etc

Outcome

1. Assessment of outcome
    a. independent blind assessment ★
    b. record linkage ★
    c. self report
    d. no description
2. Was follow-up long enough for outcomes to occur
    a. yes - US within the 1st week of life ★
    b. no
3. Adequacy of follow up of cohorts
    a. complete follow up - all subjects accounted for ★

b. subjects lost to follow up unlikely to introduce bias - small number lost - > 80 % follow up, or description provided of those lost) ★
c. follow up rate < 79% (select an adequate %) and no description of those lost
d. no statement

Newcastle Ottawa Scale: COHORT STUDIES									
	**Selection**				**Comparability**	**Outcome**			**Total**
	**Representativeness of the Exposed Cohort**	**Selection of the Non-Exposed Cohort**	**Ascertainment of Exposure**	**Demonstration That Outcome of Interest Was Not Present at Start of Study**	**Comparability of Cohorts on the Basis of the Design or Analysis**	**Assessment of Outcome**	**Was Follow-Up Long Enough for Outcomes to Occur**	**Adequacy of Follow Up of Cohorts**	
Berger, 1997	★<br>• 90% of infants born between 1984-1988 were included in the study	★<br>• Same cohort analysed	★<br>• Record based	-	★<br>• Controlled for weight	★<br>• Record linkage	-<br>• Ultrasound performed day 5-8	★<br>• ~90% of infants included	★★★★★★<br>Moderate Risk
Mercuri, 1998	-<br>• Selected group → infants on PNW deemed as 'normal'	★<br>• Same cohort analysed	★<br>• Record based	-<br>• Unknown	★<br>• Controlled for weight	★<br>• Record linkage	-<br>• No infants enrolled when 6-48 hours of life, unknown timepoint for ultrasound to be performed	-<br>~ 32% of infants lost to follow up	★★★★<br>Moderate Risk
Gilbert, 2003	★<br>• State-wide database in California used<br>• 1.4 million deliveries over a 2-year period	★<br>• All from same database	★<br>• ICD-9 codes used	-<br>• Congenital malformations not excluded from	★<br>• FGR vs non FGR	★<br>• Record linkage	-<br>• Unknown	★<br>• 1.9% of data from database excluded due to inconsiste	★★★★★★<br>Moderate Risk

	included<br>• < 2% of deliveries in California not captured in database<br>• 97.9% of reported singleton deliveries linked with records			database<br>• Given this, unlikely CUAs known antenatally would have been excluded				ncy with reported gestational age and birth weights	
Baschat, 2007	-<br>• Selected group → multicentre- 12 academic perinatal centres with tertiary NICU.<br>• Inclusion- normal fetal anatomy, AC <5th centile, Abnormal UA doppler flow, normal fetal karyotype, intact membranes without evidence of chorioamnionitis and/or perinatal infection.	-<br>• No non-exposed cohort included	★<br>• Records	★<br>• Normal fetal anatomy was an inclusion criterion	-<br>• Outcome of interest not controlled for	★<br>• Record linkage	-<br>• Unknown	-<br>• No statement	★★★<br>High Risk
Valcamonico, 2007	-<br>• Selected group → < 1000g and ≤ 34 weeks, without any morphological and/or chromosomal anomalies over a	-<br>• No non-exposed cohort	★<br>• Record based	★<br>• Congenital malformations excluded from cohort	-<br>• Outcome of interest not controlled for	★<br>• Record linkage	-<br>• Unknown	-<br>• No statement	★★★<br>High Risk

	5 year period and born in Brescia								
Marsoosi, 2012	- • Selected group- FGR infants admitted to the perinatology warn in Tehran	- • No non-exposed cohort included	★ • Record based	★ • Exclusion criteria- Intrauterine IVH and structural or chromosomal anomalies	★ • All infants given steroids	★ • Record linkage	★ • Ultrasounds performed until day 7	★ • 5% of infants did not have ultrasounds performed	★★★★★★ Moderate Risk
Ballardini, 2014	★ • All infants born at University Hospital in Italy between 33-36^{+6} weeks included	★ • Same cohort analysed	★ • NEOCARE database used	★ • Data on known malformations collected	★ • Controlled for weight <10th and < 3rd centiles	★ • Record linkage	- • Screened between 3-7th day and repeated depending on result and gestational age	★ • No infants lost to follow up	★★★★★★ ★ Low Risk
Tul, 2015	★ • National registry used with birth data collected over a 10-year period	★ • Same cohort analysed	★ • Record based	- • Unknown	★ • Controlled for weight	★ • Record linkage	- • Unknown	★ • All infants in dataset were included	★★★★★★ Moderate Risk
Starcevic, 2016	- • Selected group → 60 infants with late onset FGR	- • No non-exposed cohort included	★ • Record based	★ • Congenital anomalies excluded	- • Outcome of interest not controlled for	★ • Record linkage	★ • Ultrasounds performed on day 1,3 and 7	★ • All subjects accounted for	★★★★★ Moderate Risk

Krishnamurthy, 2017	-<br>• Selected group → Infants < 10th centile over a 4 year period in a tertiary hospital in Melbourne	-<br>• No non-exposed cohort included	★<br>• Record based	-<br>Congenital malformations not separated from results	-<br>• Outcome of interest not controlled for	★<br>• Record linkage	-<br>• Unknown	★<br>• 1% of study population excluded due to incomplete information	★★★<br>High Risk
Stimac, 2019	-<br>• Selected group → Croatian hospital database used – tertial centre which cares for population of 500, 000<br>• 7 year period of deliveries included<br>• Infants < 5th centile based on Croatian standard birth weights included	-<br>• No non-exposed cohort included	★<br>• Records-from database	-<br>• Unknown	-<br>• Outcome of interest not controlled for	★<br>• ICD-10 codes from records used	-<br>• Unknown	-<br>• No statement	★★<br>High Risk
Medina-Alva, 2019	-	-<br>• No non-exposed cohort	★<br>• Record based	★	-<br>• Outcome of interest not controlled for	★	★	-	★★★★<br>Moderate<br>Risk
Turcan, 2020	★<br>• Hospital based database (in Bucharest) over 3	★<br>• Same cohort analyse	★<br>• Record based	-<br>• Congenital malformations not	★<br>• Controlled for weight	★<br>• Record linkage	★<br>• Unknown	-<br>• No statement	★★★★★★<br>Moderate

	years	d		separated from results					Risk

One star was awarded for each domain, except for comparability, where a maximum of two starts could be awarded. Total scores ranged from 0-9. Low risk of bias studies were awarded 7-9 stars, moderate risk of bias studies were awarded 4-6 stars and high risk of bias studies were awarded 0-3 stars.

Newcastle Ottawa Scale

CASE CONTROL STUDIES

Note : A study can be awarded a maximum of one star for each numbered item within the Selection and Exposure categories. A maximum of two stars can be given for Comparability

Selection

1. Is the case definition adequate?
   a. yes, with independent validation ★
   b. yes, eg record linkage or based on self-reports
   c. no description
2. Representativeness of the cases
   a. consecutive or obviously representative series of cases ★
   b. potential for selection biases or not stated
3. Selection of Controls
   a. community controls ★
   b. hospital controls
   c. no description
4. Definition of Controls
   a. no history of disease (CUAs) ★
   b. no description of source

Comparability

1. Comparability of cases and controls on the basis of the design or analysis
   a. study controls for weight ★
   b. study controls for any additional factor ★ (Steroid use)

Exposure

1. Ascertainment of exposure
   a. secure record ★
   b. structured interview where blind to case/control status ★
   c. interview not blinded to case/control status
   d. written self-report or medical record only
   e. no description
2. Same method of ascertainment for cases and controls
   a. yes ★
   b. no
3. Non-Response rate
   a. same rate for both groups ★
   b. non respondents described
   c. rate different and no designation

Newcastle Ottawa Scale: CASE CONTROL STUDIES

	**Selection**				**Comparability**	**Exposure**			**Total**
	**Is the Case Definition Adequate**	**Representativeness of the Cases**	**Selection of Controls**	**Definition of Controls**	**Comparability of Cases and Controls on the Basis of the Design or Analysis**	**Ascertainment of Exposure**	**Same method of ascertainment for cases and controls**	**Non-Response Rate**	
Rocha, 2010	★<br>• Infants < 10th centile based on American growth charts born 34-36 weeks	-<br>• Potential for selection bias not stated	-<br>• Hospital based controls	★<br>• Infants born at the same gestation with weight between $10\text{-}89^{th}$ centile	★<br>• Study controls for weight	★<br>• Medical records used	★<br>• Retrospectively recruited for both	-<br>• No statement	★★★★★<br>★<br>Moderate Risk

One star was awarded for each domain, except for comparability, where a maximum of two starts could be awarded. Total scores ranged from 0-9. Low risk of bias studies were awarded 7-9 stars, moderate risk of bias studies were awarded 4-6 stars and high risk of bias studies were awarded 0-3 stars.
